# Influence of the Ce^4+^/Ce^3+^ Redox-Couple on the Cyclic Regeneration for Adsorptive and Catalytic Performance of NiO-PdO/CeO_2±δ_ Nanoparticles for *n*-C_7_ Asphaltene Steam Gasification

**DOI:** 10.3390/nano9050734

**Published:** 2019-05-13

**Authors:** Oscar E. Medina, Jaime Gallego, Laura G. Restrepo, Farid B. Cortés, Camilo A. Franco

**Affiliations:** 1 Grupo de Investigación en Fenómenos de Superficie—Michael Polanyi, Departamento de Procesos y Energía, Facultad de Minas, Universidad Nacional de Colombia, Sede Medellín, Medellín 050034, Colombia; oemedinae@unal.edu.co (O.E.M.); lgrestrepob@unal.edu.co (L.G.R.); 2 Química de Recursos Energéticos y Medio Ambiente, Instituto de Química, Universidad de Antioquia UdeA, Calle 70 No. 52-21, Medellín 050010, Colombia; andres.gallego@udea.edu.co

**Keywords:** adsorption, asphaltene, catalytic steam gasification, cerium redox cycle, thermal EOR regeneration cycles, nanoparticles

## Abstract

The main objective of this study is to evaluate the regenerative effect of functionalized CeO_2±δ_ nanoparticles with a mass fraction of 0.89% of NiO and 1.1% of PdO in adsorption and subsequent decomposition of *n*-C_7_ asphaltenes in steam gasification processes. During each regeneration cycle, the adsorption capacity and the catalytic activity of the nanoparticles were evaluated. To estimate the adsorption capacity of the nanoparticles, adsorption kinetics were studied at a fixed concentration of *n*-C_7_ asphaltenes of 10 mg·L^−1^ as well as adsorption isotherms at three different temperatures at 25 °C, 55 °C, and 75 °C. To evaluate the catalytic activity, the loss of mass of the nanoparticles was evaluated by isothermal conversions with a thermogravimetric analyzer at 230 °C, 240 °C, and 250 °C, and at non-isothermal conditions involving a heating from 100 °C to 600 °C at a 20 °C·min^−1^ heating rate. The asphaltenes showed a high affinity for being adsorbed over the nanoparticles surface, due to the nanoparticles-asphaltene interactions are stronger than those that occur between asphaltene-asphaltene, and this was maintained during nine evaluated regeneration cycles as observed in the Henry’s constant that increased slightly, with changes of 21%, 26% and 31% for 25 °C, 55 °C and 75 °C. Polanyi’s adsorption potential decreases by 2.6% for the same amount adsorbed from the first cycle to the ninth. In addition, the catalytic activity of the nanoparticles did not change significantly, showing that they decompose 100% of the *n*-C_7_ asphaltenes in all cycles. However, the small decrease in the adsorption capacity and catalytic activity of the nanoparticles is mainly due to the presence and change in concentration and ratio of certain elements such as oxygen, iron or others at the surface of the nanoparticle as shown by X-ray photoelectron spectroscopy (XPS) analyses. Thermodynamic parameters of adsorption such as $\Delta H_{ads}^{o}$, $\Delta S_{ads}^{o}$, and $\Delta G_{ads}^{o}$ and the effective activation energy (*E_a_*) were calculated to compare adsorptive and catalytic performance during each cycle. There is an increase of 9.3% and 2.6% in the case of entropy and enthalpy, respectively, and a decrease of 0.5%, 3.1% and 6.5% for 25 °C, 55 °C and 75 °C respectively for the Gibss free energy from cycle 1 to cycle 9. It was found that these parameters are correlated with the Ce concentration and oxidation state ratios (Ce^3+^/Ce^4+^ couple) at the surface.

## 1. Introduction

With the reduction of conventional oil reserves and the growth of global energy demand, it is necessary to restore or increase these reserves by encouraging the production of heavy (HO) and extra-heavy oils (EHO) [1,2]. However, these type of crude oils present difficulties in production, transport, and refinery, which implies an increase in costs. This is because they are characterized by a lowAmerican Petroleum Institute (API) gravity [3,4,5,6,7] and a high viscosity, due mainly to the high content of asphaltenes [8,9]. For this reason, enhanced oil recovery methods (EOR) have been developed to facilitate the extraction of HO and EHO including chemical processes such as polymer flooding and thermal processes [10,11,12]. Thermal EOR methods are divided into two groups: those that use hot fluid (water, steam) from the surface to transfer the energy and those that generate chemical reactions in crude oil during the injection process such as in-situ combustion (ISC). In particular, steam injection is performed by continuous or cyclic injection [13] mainly focused on oil viscosity reduction and thermal expansion. Cyclic steam injection is the most used mechanism due to its easy implementation in the field. It consists of a single well operation where the steam is injected over a certain time [14]. This is followed by the soaking stage (from one to three weeks), where the energy contained in the steam is transferred to the formation and the reservoir fluids. After, the well is opened for production [15]. When production declines to such a point that it ceases to be profitable, a second cycle can be carried out to increase production. In this way, several cycles can be performed until it is not profitable for the production that is projected [16].

However, this method involves certain problems. As steam injection cycles are performed, the lighter components of the crude oil matrix are vaporized [17]. The produced crude oil undergoes an increase in the cracking activation energy, and a heavier residual oil is generated due to the loss of the light components [18,19]. Other problems associated with this technique include the decrease in performance as the steam is injected cyclically and the operating temperature ranges do not exceed 300 °C [16]. Hence, permanent improvement in the oil quality cannot be achieved since the asphaltene compounds start their decomposition around 450 °C in a steam atmosphere [13,20,21].

Consequently, some catalytic agents such as nanoparticles have been used for the adsorption [21,22,23,24,25] and subsequent transformation of the asphaltenes [26,27,28,29,30,31]. This is due to nanoparticle properties, such as size, surface area, and surface energy density, among others, that favors the application to the HO and EHO reservoirs [13,20,21,25,31,32,33,34,35,36,37,38]. Some nanoparticles that have been used in steam gasification [25] are derived from transition element oxides, such as nanoparticles of Co_3_O_4_, NiO, Fe_3_O_4_ [20]. Also, there are functionalized materials supported on silica [13], alumina [39], ceria [40], titania [20], among others [41,42,43]. In these studies, it was found that the catalytic activity is affected by the nature of the oxide and the interactions that occur between asphaltenes and nanoparticles, and it was shown that with the presence of the nanoparticles the activation energy significantly decreases [8,44,45]. Also, it was found that the functionalization of the nanoparticles improves their catalytic behavior by enhancing the heavy oil compounds decomposition [40].

The implementation of nanocatalysts promotes the generation of several reactions, such as CO_2_ reduction, water-gas shift [46], methanization [47], partial oxidation [48], Boudouard [49] and steam reforming reactions [28,34,50,51]. Maintaining the reaction rate during steam injection cycles requires catalysts capable of being regenerated in-situ and having an appropriate half-life. Due to the redox behavior of CeO_2±δ_, this material has regenerative autocatalytic properties which means that the result after any catalytic process is the same as initially, where one of the components of the reaction is the one that acts as a catalyst [52]. This will allow it to reverse the oxidation state or the loss of an electron, from Ce^4+^ to Ce^3+^, indicating that it would return to its initial conditions, allowing the nanoparticles to be reutilized and making possible a better use of their properties. The catalytic character of the redox cycle (Ce^3+^/Ce^4+^) has been demonstrated in reactions such as CH_4_/CO_2_ reforming [53]. However, until now, the application of this material as a supporting agent or catalyst for its regeneration in adsorption-decomposition cycles of heavy compounds has not been reported.

This property of CeO_2±δ_ is due to the changes in oxidation state according to the stimuli to which it is subjected. This behavior allows it to participate in several chemical reactions, which in turn allow it to reverse its oxidation state, returning it to its initial state [54]. Another proposed mechanism for the ceria regeneration and its return to the initial state is proposed by Das et al. (2007), who suggest that the mixture of valence states on the surface of CeO_2±δ_ nanoparticles acts as an antioxidant, allowing it to release free radicals [55].

Furthermore, the addition of transition element oxides, such as NiO, and noble element oxides, such as PdO, can improve the catalytic performance of the support through a synergetic effect known as strong metal support interaction phenomena (SMSI) [56]. Other studies show that ceria as support has a synergistic effect with nickel highly dispersed in zeolite socony moil-five (IFM) structures, reducing the start temperature of the water gas shift (WGS) reaction to low temperatures of around of 230 °C [57]. As for Pd, it increases the interactions between the support and the gases produced through the production of formiate species. When ceria returns to its initial state, hydrogen release reactions and a generation of free radicals will occur again, which indicates that a cycle of regeneration or autocatalytic reaction has been developed. This could allow the conservation of its capacity of *n*-C_7_ asphaltene adsorption and decomposition, while the heavy molecules could become smaller as each cycle occurs and could decrease the problems related to heavy and extra heavy oils. For this reason, through experiments of adsorption and thermogravimetric analysis, the affinity for asphaltenes and catalytic activity of NiO and PdO nanoparticles supported on CeO_2±δ_ were designed [41]. In the development of this study, the loads of the transition element oxides (TEO) on the support were optimized to generate a greater asphaltene conversion with steam present at low temperatures (220 °C), and it was found that with a mass fraction of 0.89% and 1.1% of NiO and PdO, respectively, it was possible to maximize the conversion of the asphaltenes to 100% in less than 80 min.

It is worth mentioning that, to the best of our knowledge, there has not been any report in the scientific literature of nanoparticle regeneration (adsorption/steam gasification cycles) for thermal enhanced oil recovery processes. Therefore, the main objective of this work was to evaluate the regenerative capacity of CeO_2±δ_ nanoparticles with the optimal loads on their surface through the evaluation of several cycles of *n*-C_7_ asphaltene adsorption/catalysis in a steam gasification process. The evaluation has taken into account the influence of the redox cycle (Ce^3+^/Ce^4+^) on the self-regeneration of CeO_2±δ_. This work will open a new approach for future studies on the application of nanocatalysts in regenerative processes during EOR applications.

## 2. Materials and Methods

### 2.1. Materials

The *n*-C_7_ asphaltenes were extracted from an extra heavy Colombian crude oil using an excess amount of *n*-heptane (99%, Sigma-Aldrich, St. Louis, MO, USA) in a volume ratio of 40:1. Toluene (99.5%, Merk KGaA, Darmstadt, Germany) was used to prepare asphaltene solutions for adsorption experiments. The crude oil has a viscosity of 3.1 × 10^6^ cP at 25 °C and 6.4°API, and an approximate content of saturates, aromatics, resins and asphaltenes of mass fraction of 13.0%, 16.9%, 49.9%, and 20.2%, respectively. Ceria (CeO_2_) nanoparticles of 21.6 nm and a surface area of 65 m^2^·g^−1^ ± 2 m^2^·g^−1^ were purchased from Nanostructured and Amorphous Materials (Houston, TX, USA) and were used as support [58,59]. The nanoparticles were functionalized with a mass fraction of 0.89% and 1.1% of NiO and PdO, respectively, using the incipient wetness technique. These amounts of NiO and PdO have been optimized in a previous study [40]. The sample was labeled CeNi0.89Pd1.1 and had an Ni–Pd particle size of 5.53–3.61 nm with a dispersion of 25% and 36%, respectively. A more detailed description of the characterization of the nanoparticles used in the present study can be found in a previous study [40].

### 2.2. Methods

#### 2.2.1. Selection of Catalytic Nanoparticles

For the selection of nanocatalyst, a series of tests have been carried out previously, starting from the evaluation of three bimetallic systems supported on CeO_2±δ_ nanoparticles functionalized with transition element oxides (TEO) for the pairs Ni–Pd, Co–Pd and Fe–Pd [40]. Performance was studied according to adsorption isotherms, thermogravimetric analysis, isothermal conversions, gases produced, and coke yield. Then, a three-component simplex centroid mixture design (SCMD) was performed, varying the dosage of the TEO on the surface of the nanoparticle from a mass fraction of 0% to 2.0%. The nanoparticles were functionalized with a mass fraction of 0.89% and 1.1% of NiO and PdO, respectively.

#### 2.2.2. Nanoparticles Regeneration

The nanoparticles regeneration process starts from the asphaltene adsorption followed by their catalytic decomposition. The nanoparticles with adsorbed asphaltenes were subjected to a steam atmosphere in a tubular furnace (Thermo Scientific Lindberg/Blue, Waltham, MA, USA) at 240 °C for 2 h. For this, N_2_ flow was fixed at 100 mL·min^−1^ and steam injection was performed at a flow rate of 6.3 mL·min^−1^ using a gas saturator with controlled temperature. In each regeneration cycle, part of the nanoparticles was taken to perform the thermogravimetric and X-ray photoelectron spectroscopy analyses.

#### 2.2.3. The *n*-C_7_ Asphaltene Adsorption over Nanoparticles

For the adsorption tests, a stock solution containing 15,000 mg·L^−1^ of *n*-C_7_ asphaltenes in toluene was prepared and subsequently diluted to different concentrations. The changes in concentration after adsorption were determined by UV-vis spectrophotometer (Thermo Scientific, Waltham, MA, USA). The initial concentrations of the *n*-C_7_ asphaltene solutions varied from 100 mg·L^−1^ to 1500 mg·L^−1^. A fixed amount of nanoparticles (100 mg per 10 mL of model solution) was employed. Once the nanoparticles were added to the solutions, they were stirred at 300 rpm and an aliquot was taken every 10 min for evaluating adsorption kinetics until the amount adsorbed remained constant. The nanoparticles with *n*-C_7_ asphaltenes adsorbed were separated by centrifugation at 5000 rpm for 45 min and dried in a vacuum oven at 60 °C for 24 h. The adsorption experiments were carried out at temperatures of 25 °C, 55 °C, and 75 °C. The amounts adsorbed in units of mg of *n*-C_7_ asphaltenes per gram mass of nanoparticle was estimated according to Equation (1).

(1) $$N_{ads}=\frac{C_{o}-C_{E}}{W}V,$$

where $C_{o}$ (mg·L^−1^) and $C_{E}$ (mg·L^−1^) are the initial and equilibrium concentrations in the equilibrium, respectively; V (L) is the volume of the solution and W (g) is the mass of nanoparticles added to the solution. The concentration of *n*-C_7_ asphaltenes in the supernatant was measured at a wavelength of 298 nm using a Genesys 10S UV-vis spectrophotometer with an uncertainty of 0.001 a.u. in the measurement of absorbance. This implies a standard deviation of 0.05 mg·L^−1^ in the calculation of the residual concentration.

#### 2.2.4. Thermogravimetric Analyses

The catalytic activity of nanoparticles in the steam gasification of the *n*-C_7_ asphaltenes adsorbed on the nanoparticle surface was evaluated using a thermogravimetric analyzer Q50 (TA Instruments, Inc., New Castel, DE, USA). For the development of the tests, N_2_ flow was fixed at 100 mL·min^−1^ and hauling steam controlled by thermostatic bath was introduced at the same time at a flow rate of 6.3 mL·min^−1^ using a gas saturator. During each catalytic regeneration cycle, the nanoparticles with adsorbed *n*-C_7_ asphaltenes were subjected to the TGA under isothermal and non-isothermal conditions. For tests at isothermal conditions, the samples were heated to three different temperatures (230 °C, 240 °C, and 250 °C) [21]. On the other hand, the experiments at non-isothermal conditions were performed by heating the samples from 100 °C to 600 °C at a heating rate of 20 °C·min^−1^ to observe the rate for mass loss of *n*-C_7_ asphaltenes on the nanoparticle surface [60]. Finally, each run at isothermal conditions was carried out with an adsorbed amount of *n*-C_7_ asphaltenes that remained constant at 0.02 mg·m^−2^, while for non-isothermal conditions the asphaltene load was 0.2 mg·m^−2^.

#### 2.2.5. X-ray Photoelectron Spectroscopy Analysis

During each catalytic regeneration cycle, the nanoparticles were characterized by X-ray photoelectron spectroscopy (XPS) using a Specs X-ray photoelectronic spectrometer (NAP-XPAS) using a monochromatic source of Al−Kα (1486.7 eV, 13 kV, 100 W) together with a PHOIBOS 150-1D-DLD analyzer. Energies of 100 eV (1 eV·step^−1^) during three measurement cycles and 20 eV (0.1 eV·step^−1^) in ten measurement cycles were used for general and high-resolution spectra, respectively.

## 3. Modeling

### 3.1. Double Exponential Model

In this model, the adsorption mechanism and the transfer of the adsorbate to the adsorbent are described by two steps. In the first step, there is a fast transfer from the bulk to the adsorbent. This is followed by a second step, which is rate-determining, and is dominated by the diffusion through the adsorbent surface and asphaltene self-association over the active sites until equilibrium is reached. Equation (2) describes this model.

(2) $$N_{ads}=\;N_{ads,e}-\frac{D_{f}}{V}\exp\left(-k_{f}t\right)-\frac{D_{s}}{V}\exp\left(-k_{s}t\right),$$

where the amount adsorbed at time t is expressed by $N_{ads}$ (mg·g^−1^), the amount adsorbed in the equilibrium and the volume ratio of solvent/mass of material added to the adsorptive processes carried out are $N_{ads,e}$ (mg·g^−1^) and V (L·g^−1^). Additionally, the adsorption and mass transfer coefficients for the fast stage are given by $D_{f}$ (mg·L^−1^) and $k_{f}$ (min^−1^) respectively, while the adsorption and mass transfer coefficients for the slow stage are $D_{s}$ (mg·L^−1^) and $k_{s}$ (min^−1^). If $k_{f}>>k_{s}$, the exponential term corresponding to the rapid process can be assumed to be negligible [61,62,63].

### 3.2. Solid−Liquid Equilibrium (SLE) Model

The chosen model allows the description of the adsorption isotherms using the theory of association and adsorption of molecules in micropores suggested by Talu and Meunier [64]. The model can be described by Equations (3)–(5): [65]

(3) $$C=\frac{\psi K_{H}}{1+K\psi }e^{\left(\frac{\psi }{A\cdot N_{m}}\right)}$$

(4) $$\;\psi =\frac{-1+\sqrt{1+4K\xi }}{2K}$$

(5) $$\xi =\;\frac{N_{m}N}{\left(N_{m}-N\right)},$$

where C (mg·g^−1^), represents the concentration of *n*-C_7_ asphaltenes in equilibrium and $K_{H}$ (mg·g^−1^) is the affinity of the adsorbate for the surface of the solid. A low value implies greater affinity, i.e., greater accessibility (or ease of access) of the *n*-C_7_ asphaltenes to the active sites.

Likewise, K (g·g^−1^) is a constant related to the asphaltene self-association over the active sites on the surface of the nanoparticles, $N_{m}$ (g·g^−1^) is the maximum capacity of *n*-C_7_ asphaltene adsorption and the amount adsorbed is expressed as N (g·g^−1^). Finally, ξ is a relation that depends on the maximum adsorption capacity ($N_{m}$) and amount adsorbed (N).

### 3.3. Thermodynamic Properties of Adsorption

To better understand the effect of temperature on *n*-C_7_ asphaltene adsorption, thermodynamic parameters are used. To describe the thermodynamic properties of adsorption, five temperature-independent parameters are needed, using equations of the SLE model, and replacing $K_{H}$ and K with Equations (6) and (7) shown below.

(6) $$K_{H}=e^{\left(H_{0}+\frac{H_{1}}{T}\right)}$$

(7) $$\;K=e^{\left(K_{0}+\frac{K_{1}}{T}\right)},$$

where $K_{0}$, is related to the entropy of reaction and $K_{1}$, related to enthalpy. It is possible to find the three thermodynamic parameters, change in entropy $\Delta S_{ads}^{o}$, change in enthalpy $\Delta H_{ads}^{o}$ and change in Gibbs free energy $\Delta G_{ads}^{o}$, through Equations (8)–(10).

(8) $$\Delta S_{{{}_{a}}_{ds}}^{o}=K_{0}R$$

(9) $$\Delta H_{{{}_{a}}_{ds}}^{o}=K_{1}R$$

(10) $$\Delta G_{{{}_{a}}_{ds}}^{o}=-RT\ln K.$$

A negative value for the Gibbs free energy change would indicate the spontaneity and thermodynamic favorability of the process. Similarly, a negative enthalpy change value would suggest that the process occurs exothermically; negative enthalpy also suggests that the adsorption would decrease as temperature increased.

Finally, a positive value of the entropy change would indicate that due to the *n*-C_7_ asphaltene adsorption, there would be an increase in the randomness of the system at the liquid-solid interface (nanoparticle *n*-C_7_ asphaltenes) [65,66].

### 3.4. Adsorption Potential Model by Michael Polanyi

The adsorption potential is defined as the work required for an adsorbate molecule to be transferred from the bulk phase to the surface of the adsorbent. In this theory, it is considered that the adsorbed layer is a thick film, whose density decreases as the distance from the surface increases. It is independent of the temperature for a fixed adsorbed amount $N_{ads}$. The adsorption potential or Polanyi’s potential can be expressed as shown in Equation (11):

(11) $$A=RT\ln\left(1+\frac{1}{C_{E}}\right),$$

where the adsorption potential is represented by A, and the concentration of *n*-C_7_ asphaltenes in equilibrium is expressed as $C_{E}$ (g·g^−1^). Additionally, R (J·mol^−1^·K^−1^) is the constant of ideal gases, and T (K) is the system absolute temperature [67,68].

### 3.5. Estimation of Effective Activation Energy

The calculation of the activation energy was carried out with the following model given by the ICTAC Kinetics committee. Equation (12) provides a basis for differential kinetic methods and applies to any temperature [69,70].

(12) $$\frac{d\alpha }{dt}=K_{\alpha }\exp\left(-\frac{E_{\alpha }}{RT}\right)f\left(\alpha \right),$$

where the effective activation energy is expressed by $E_{\alpha }$ (kJ·mol^−1^), while α is the degree of conversion. The latter depends on the initial mass of the sample, the current mass at time t and, the final mass.

Likewise, $K_{\alpha }$ (s^−1^) is the pre-exponential factor and T (K) is reaction temperature. The reaction mechanism is given by f(α), and the reaction rate is dα/dt. With the analysis of isothermal conditions and integration by separation of variables, the integral reaction model given by Equation (13) is obtained:

(13) $$g(\alpha )=K_{\alpha }e^{(-\frac{E_{\alpha }}{RT})t}\;.$$

Taking constant the energy of activation and applying natural logarithm to both sides, Equation (14) is obtained,

(14) $$\ln(t_{a,i})=\ln(\frac{g(\alpha )}{K_{\alpha }})+\frac{E_{\alpha }}{RT_{i}}\;.$$

From the plot of $\ln(t_{a,i})$ vs. $1/T_{i}$, it is possible to obtain the value of the activation energy from the slope.

### 3.6. Statistical Analysis

The accuracy of the parameters for each model was presented using the optimized gradient model. The root mean square error (RMS) was selected to minimize the differences between the experimental values and the values obtained under the theoretical considerations of the different models. The values obtained for the mean squared error were calculated under Equation (15), where *m* represents the amount of data or measurements made and × the evaluated parameter for each model:

(15) $$RMS\%=\sqrt{\sum_{i}^{m}(X_{\exp erimental,i}-X_{\mathrm{mod}el,i}{)}^{2}\times m^{-1}}\times 100.$$

## 4. Results and Discussion

### 4.1. Selection of Nanocatalyst

In a previous study [40], three main systems were evaluated, the first consisting of a mass fraction of 1% of NiO and 1% of PdO (CeNi1Pd1), the second 1% of Fe_2_O_3_ and 1% of PdO (CeFe1Pd1) and the third 1% Co_3_O_4_ and 1% PdO (CeCo1Pd1), all supported on ceria nanoparticles. For the adsorption, the system CeNi1Pd1 showed better results than CeFe1Pd1 and CeCo1Pd1. The catalytic activity of the nanoparticles was evaluated with a series of tests, (a) rate for mass loss, (b) isothermal conversions, (c) analysis of the gaseous products and (d) coke yield. On all four tests, CeNi1Pd1 performed better than the other systems. Finally, to optimize the concentration of the metals of the best system, a simplex-centroid mixture design (SCMD) was made using the STATGRAPHICS Centurion XVI software (StartPoint Technologies Inc. Addison, TX, USA) [71]. This new system was called CeNi0.89Pd1.1 and had a mass fraction of 0.89% and 1.1% of NiO and PdO respectively, on the surface of the cerium oxide [40].

### 4.2. Adsorption Kinetics

**Figure 1:**
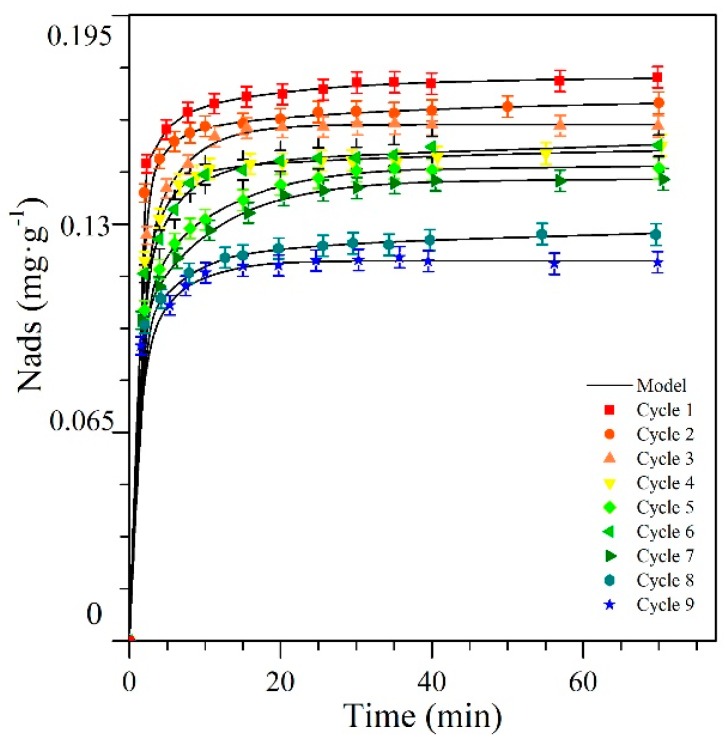
Asphaltene adsorption kinetics on CeNi0.89Pd1.1 nanoparticles through several catalytic regeneration cycles of adsorption and subsequent catalytic steam gasification. Adsorption kinetics were constructed for a fixed initial concentration of *n*-C_7_ asphaltene of 10 mg·L^−1^. The symbols are experimental data, and the continuous lines are from the double exponential model.

Figure 1 shows the adsorption kinetics for *n*-C_7_ asphaltenes on the CeNi0.89Pd1.1 nanoparticles through the different catalytic regeneration cycles at an initial concentration of 10 mg·L^−1^ of *n*-C_7_ asphaltenes. In general, two stages are observed in adsorption kinetics. The first stage is characterized by rapid adsorption due to the electrostatic and van der Waals attraction forces between the nanoparticle and the *n*-C_7_ asphaltene molecules, and in the second stage, a slow uptake is observed that involves a gradual *n*-C_7_ asphaltene adsorption on the surface of the nanoparticles through complex reactions and the subsequent self-association over the active sites [72]. From Figure 1 it is observed that the process of asphaltene adsorption is a fast process where a maximum time of 40 min is required for the equilibrium to be reached. This is due to the impediment for diffusion through the materials, related to the auto-associative characteristics of the asphaltenes, which generate growth in the size of the asphaltene according to the concentration in the system [73]. Additionally, in each cycle the time to reach equilibrium does not change drastically, and the amount of *n*-C_7_ asphaltenes that adsorbs at that point decreases from 0.1740 mg·g^−1^ to 0.1160 mg·g^−1^ from cycle one to cycle nine, respectively.

Table 1 presents the parameters of the double exponential model and the associated error obtained for the materials as a function of the initial concentration of *n*-C_7_ asphaltenes and the regeneration cycles of nanoparticles. As shown with the estimation of the associated errors, the double exponential model shows a good correlation with the experimental results. In general, a proportional relationship is not observed between the $D_{f}$ parameter and the amount adsorbed through the cycles.

The amount adsorbed can be explained by materials that, due to their high affinity for *n*-C_7_ asphaltenes, saturate quickly. This is because, with the passage of catalytic regeneration cycles, there was a loss of accessible active sites that allowed greater adsorption of the aggregates on its surface. Also, due to the low values of the parameter $K_{s}$ and $D_{s}$ it is concluded that the adsorptive process of *n*-C_7_ asphaltenes on nanoparticles CeNi0.89Pd1.1 was essentially governed by one stage [74].

### 4.3. Adsorption Isotherms

**Figure 2:**
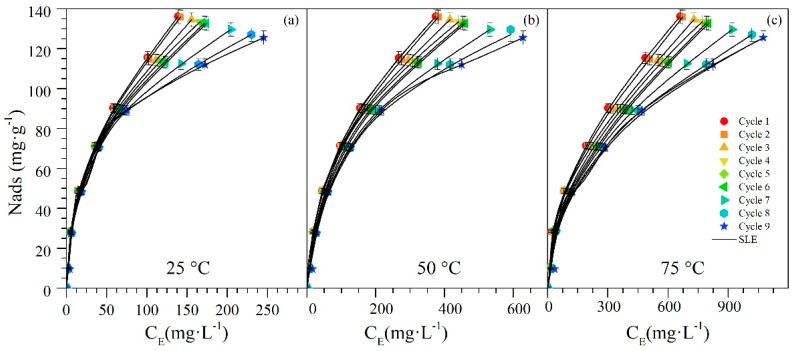
Adsorption isotherms of *n*-C_7_ asphaltenes onto CeNi0.89Pd1.1 nanoparticles evaluated at (**a**) 25 °C, (**b**) 55 °C, and (**c**) 75 °C through catalyst regeneration cycles of adsorption and subsequent catalytic steam gasification. Adsorption isotherms were constructed for different *n*-C_7_ asphaltene concentrations from 100 mg·L^−1^ to 1500 mg·L^−1^. The symbols are experimental data, and the solid lines are from the SLE model.

In Figure 2a–c, the *n*-C_7_ asphaltene adsorption isotherms are showed for the CeNi0.89Pd1.1 nanoparticle at 25 °C, 55 °C, and 75 °C through several cycles of *n*-C_7_ asphaltene adsorption. It was observed that these nanoparticles have a high amount of adsorbed *n*-C_7_ asphaltenes and this feature is maintained in subsequent cycles. For all cases, type Ib isotherms were obtained according to the IUPAC classification. The effect of temperature, the experimental results, and the parameter $N_{max}$ of the SLE model show that with increasing temperatures, the adsorbed amount of *n*-C_7_ asphaltenes decreases [73]. This is due to the temperature influence on the aggregation state of the *n*-C_7_ asphaltenes to the extent that the latter can be adsorbed in different forms such as aggregates, or individual molecules [73]. On the other hand, Figure 2 shows that the adsorption isotherms obtained throughout the cycles remain type Ib, which means that the high affinity of the *n*-C_7_ asphaltenes towards the nanoparticles is maintained. In addition, at low concentrations (C_o_ < 500 mg·L^−1^) the amount adsorbed does not change significantly in any of the cycles evaluated. However, the values of the maximum possible adsorbed amounts were reduced from 27.03 × 10^−2^ g·g^−1^, 28.86 × 10^−2^ g·g^−1^, and 29.68 × 10^−2^ g·g^−1^ for cycle 1 to 22.37 × 10^−2^ g·g^−1^, 24.03 × 10^−2^ g·g^−1^, and 25.36 × 10^−2^ g·g^−1^ for cycle 9 at 25 °C, 55 °C, and 75 °C, respectively.

Table 2 presents the parameters of the SLE model for the *n*-C_7_ asphaltenes adsorbed on nanoparticles. No significant change of $K_{H}$ is observed as the cycles occur. This value increased by approximately 21%, 26% and 31% for 25 °C, 55 °C and 75 °C respectively from cycle 1 to cycle 9, meaning that the adsorption affinity is strongly affected by the regeneration cycles. On the other hand, the K parameter did not exhibit great changes (from 1.15 × 10^−2^ to 1.17 × 10^−2^), and this means that the degree of slef-association of *n*-C_7_ asphaltenes tends to stay constant with the passage of the regeneration cycles.

### 4.4. Thermodynamic Studies

Table 3 summarizes the values of the thermodynamic parameters calculated from the effect of temperature on *n*-C_7_ asphaltene adsorption onto CeNi0.89Pd1.1 nanoparticles. In all cases the negative value of $\Delta G_{ads}^{o}$ was maintained throughout the 9 cycles of *n*-C_7_ asphaltene adsorption/decomposition, indicating that no additional energy was necessary to generate the interactions between the asphaltene molecules and the nanoparticles. This indicates that the process was thermodynamically favorable. In addition, $\Delta G_{ads}^{o}$ decreased only from cycle 1 to cycle 9 by 0.5%, 3.1%, and 6.5% for 25 °C, 55 °C, and 75 °C, respectively, suggesting that the reaction did not lose spontaneity. Contrarily, the values $\Delta S_{ads}^{o}$ were positive in all cases and these do not exhibit a considerable increase with each regeneration cycle. Therefore, the randomness at the interface of the adsorbent/adsorbate did not change. It is important to note here that its value only increased by 9.3% in cycle 9 concerning adsorption cycle 1. This slight increase in $\Delta S_{ads}^{o}$ could be due to once the asphaltene is adsorbed on the nanoparticle, the self-association over the active sites is promoted. The values of the $\Delta H_{ads}^{O}$ were negative for all cycles evaluated, suggesting the exothermic nature of the interactions between the *n*-C_7_ asphaltenes and the surface. This value increased only 2.6% from cycle 1 to cycle 9. The evaluated thermodynamic parameters were in agreement with Franco et al. [75] and Nassar et al. [66].

### 4.5. Polanyi’s Adsorption Potential

**Figure 3:**
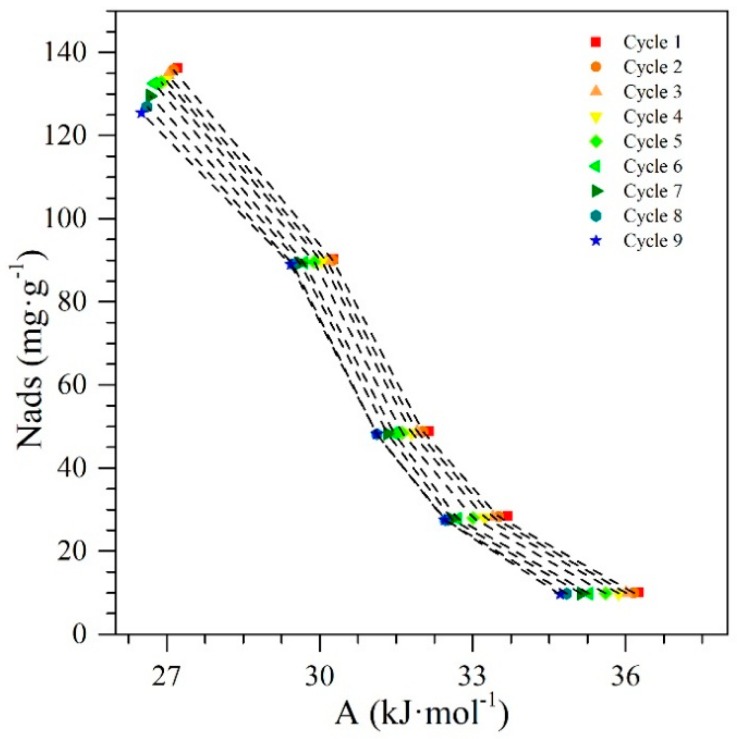
Polanyi’s adsorption potential (*A*) as a function of the amoun adsorbed (*N_ads_*) for *n*-C_7_ asphaltene adsorption on CeNi0.89Pd1.1 nanoparticles through several catalyst regenerations of adsorption and subsequent catalytic steam gasification.

The characteristic curves for the *n*-C_7_ asphaltene adsorption over the surface of the nanoparticles are illustrated in Figure 3. It is important to mention at this point, that a low value of the Polanyi’s potential implies the need for a lower energy demand to carry out the adsorptive phenomenon. As it can be observed, the adsorption potential slightly decreases as more cycles have occurred. However, it did not decrease considerably from cycle 1 to cycle 9, due to the adsorptive capacity of the nanoparticles is conserved and the adsorption remains strong during all cycles evaluated. In other words, the work required to transfer an asphaltene molecule from the surface to a given distance from the surface did not vary significantly, decreasing by only 2.6% for the same amount adsorbed from the first cycle to the ninth. This behavior also suggests that the adsorbate−adsorbent interactions in the case of CeNi0.89Pd1.1 nanoparticles are stronger for the first cycle than for the remained. The adsorption potential corresponds, therefore, to an increase in free energy experienced by the adsorbate during adsorption at equilibrium, with its adsorbed amount at concentration $C_{E}$, indicating that the adsorbate−adsorbent interactions remain strong and the rate at with which *n*-C_7_ asphaltenes were adsorbed did not change. These results were in agreement with those found by Cortés et al. [67], Betancur et al. [68] and Wu et al. [76], who observed that a stronger affinity between the adsorbate−adsorbent pair generates a higher potential value, i.e., the adsorbate is more likely to migrate to the surface of the nanoparticle instead than remaining in the bulk phase.

### 4.6. Thermogravimetric Analysis of n-C_7_ Asphaltenes

#### 4.6.1. Mass Loss Analysis

**Figure 4:**
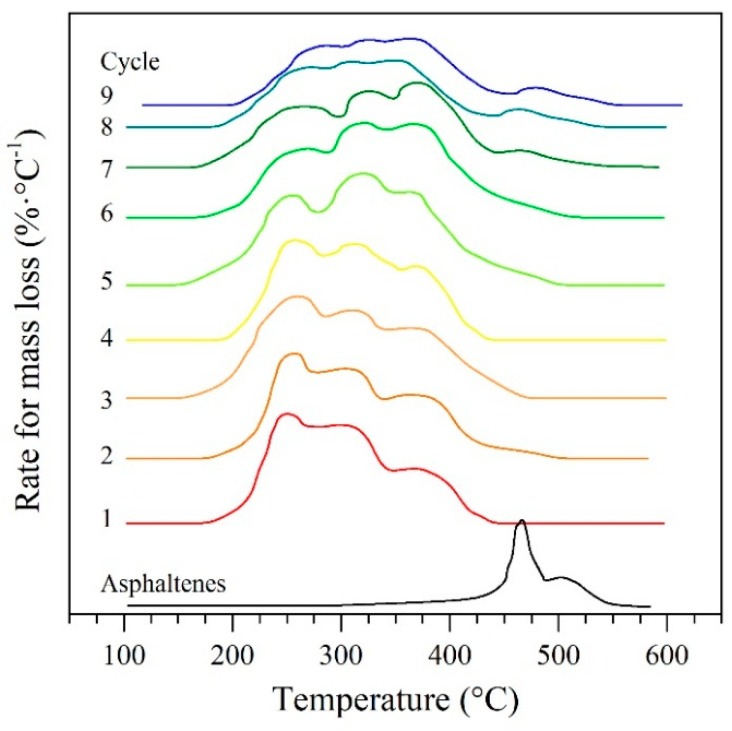
The rate for mass loss as a function of the temperature for catalytic steam decomposition of *n*-C_7_ asphaltenes in the absence and presence of CeNi0.89Pd1.1 nanoparticles for several catalyst regeneration cycles of adsorption and subsequent catalytic steam gasification. Nitrogen flow rate = 100 mL·min^−1^, H_2_O_(g)_ flow rate = 6.30 mL·min^−1^, heating rate = 20 °C·min^−1^, and asphaltene load of 0.02 mg·m^−2^.

The decomposition-gasification of *n*-C_7_ asphaltenes adsorbed on the nanoparticle CeNi0.89Pd1.1 was evaluated by heating the samples from 100 °C to 600 °C at a heating rate of 20 °C·min^−1^ under a water-saturated N_2_ atmosphere. Figure 4 shows the complete TGA profile as a function of the temperature for *n*-C_7_ asphaltenes in the absence and presence of raw and regenerated CeNi0.89Pd1.1 nanoparticles. The temperature range evaluated was divided into three main regions of low (LTR), medium (MTR) and high (HTR) temperature, as suggested in the literature [77]. The first region ends at about 250 °C, the MTR region will then range between 251 °C and 450 °C, and finally, the HTR region has temperature values between 451 °C to 600 °C.

The decomposition of *n*-C_7_ asphaltenes in the absence of nanoparticles was carried out in the HTR region, with the main decomposition at 455 °C. The presence of the CeNi0.89Pd1.1 nanoparticles as catalytic agent decreases this value to 210 °C in the LTR region, and with continuous decomposition in the MTR region and main intensities at 280 °C and 370 °C. This is due in the first instance to the heterogeneous surface of the nanoparticulate material, which due to the presence of NiO and PdO on the support, generate different catalytic effects by the different interactions between metal oxides and the support NiO/CeO_2±δ_, PdO/CeO_2±δ_, NiO-PdO/CeO_2±δ_. This could be due to the growth inhibition of PdO nanocrystals by the migration and coalescence behavior of the NiO atoms over CeO_2_ and the higher dispersion of PdO nanocrystals have concerning the NiO [78,79]. Also, a possible variation in the size of the asphaltene molecules can affect the decomposition. It is worth to remember that, as a fraction, asphaltene do not consider only one kind of molecule. Instead, a wide distribution of asphaltene molecules with diverse molecular weight can be found in the crude oil from low molecular weight samples with a high amount of aliphatic chains, to high molecular weight compounds with a large polycyclic aromatic hydrocarbons (PAH) core. This trend was maintained during all the catalytic cycles evaluated, with the difference that the intensities in the first peak decrease as the nanoparticles are regenerated and the intensities of the other two peaks increase, evidencing the suppression of the addition reactions of the *n*-C_7_ asphaltenes due to the stabilization of free radicals by hydrogen molecules in hydrogenation reactions [80,81,82]. Additionally, cycles 8 and 9 also exhibited a mass loss in the HTR region at about 455 °C. This behavior reflects that there is a loss, to a small extent, of the catalytic capacity of the nanoparticles to break down the heavier hydrocarbon chains into lower molecular weight hydrocarbons. This also lead to an increase in heteroatoms, like oxygen, and metals, such as iron, on the surface of the nanoparticle by 9.2% and 6.3% respectively (see XPS analyses below). The increase in the concentration of these elements could increase the probability of generating coordinated bonds (HA-TE), which require greater energy to break. Additionally, as explained in Section 4.7 below, CeNi0.89Pd1.1 nanoparticles lose catalytic capacity through a decrease of their concentration by approximately 28.6% of Ce^3+^ ions on the surface.

#### 4.6.2. Isothermal Conversion

**Figure 5:**
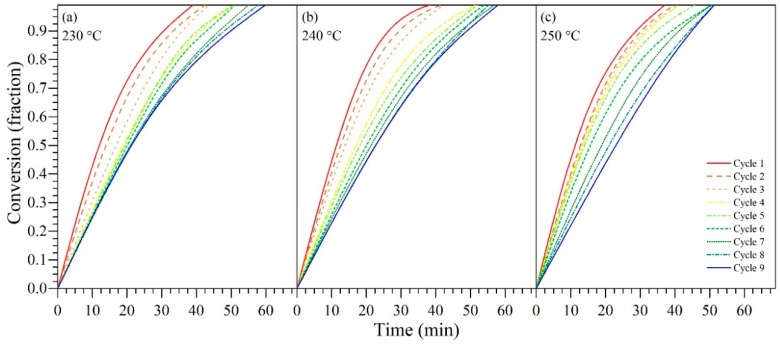
Isothermal conversion as a function of time at (**a**) 230 °C, (**b**) 240 °C, and (**c**) 250 °C for *n*-C_7_ asphaltenes in the absence and presence of CeNi0.89Pd1.1 through several catalyst regeneration cycles of adsorption and subsequent catalytic steam gasification. Nitrogen flow rate = 100 mL·min^−1^, H_2_O_(g)_ flow rate = 6.30 mL·min^−1^, heating rate = 20 °C·min^−1^, and asphaltene load of 0.2 mg·m^−2^.

Panels a–c in Figure 5 show the fraction of conversion (α) for *n*-C_7_ asphaltenes adsorbed on CeNi0.89Pd1.1 nanoparticles as a function of time at three different temperatures of 230 °C, 240 °C, and 250 °C. From Figure 5, in the first instance, it is observed that the degree of reaction (α) of the *n*-C_7_ asphaltenes in the presence of the nanoparticles changes with temperature. In general, with the increase of this variable, the reaction rate increases, and therefore, the transformation of the *n*-C_7_ asphaltene molecules into lighter hydrocarbons and consequent gas generation occur in a shorter time. This agrees with the results reported by Nassar et al. [69], Cardona et al. [39], and Medina et al. [40]. Also, the catalytic effect of the nanoparticles is reflected throughout all cycles evaluated, as CeNi0.89Pd1.1 nanoparticles are capable of decomposing 100% of *n*-C_7_ asphaltenes adsorbed. However, the time required to perform 100% of said conversion increased by 30%, 25%, and 12.5% for 230 °C, 240 °C, and 250 °C respectively, from cycle 1 to cycle 9. This behavior is corroborated by the activation energy values obtained, which increased from 10.7 kJ∙mol^−1^ to 59.8 kJ∙mol^−1^, meaning the nanoparticles required more energy for the cracking reactions. The total conversion of *n*-C_7_ asphaltenes was related, among many factors, to the role played by the hydrogen (H_2_) produced by the asphaltene-nanoparticle interactions [82]. The CeO_2±δ_ nanoparticle and the transition element oxides cracked the heavier molecules into lighter molecules, and through hydrogen production, the nanoparticles also stabilize the free radicals, which facilitates and gives support to the complete decomposition of the heavy molecules. Stabilizing the free radicals also inhibited the self-association of *n*-C_7_ asphaltenes, generating a residual of 0% [40,83,84]. The variation of the time required to convert 100% of *n*-C_7_ asphaltenes was mainly due to a loss of available active sites on the surface of the support, due to the retention of heteroatoms and metals that require a longer time to decompose.

#### 4.6.3. Effective Activation Energy of *n*-C_7_ Asphaltene Thermo-Decomposition in the Presence and Absence of Nanoparticles

**Figure 6:**
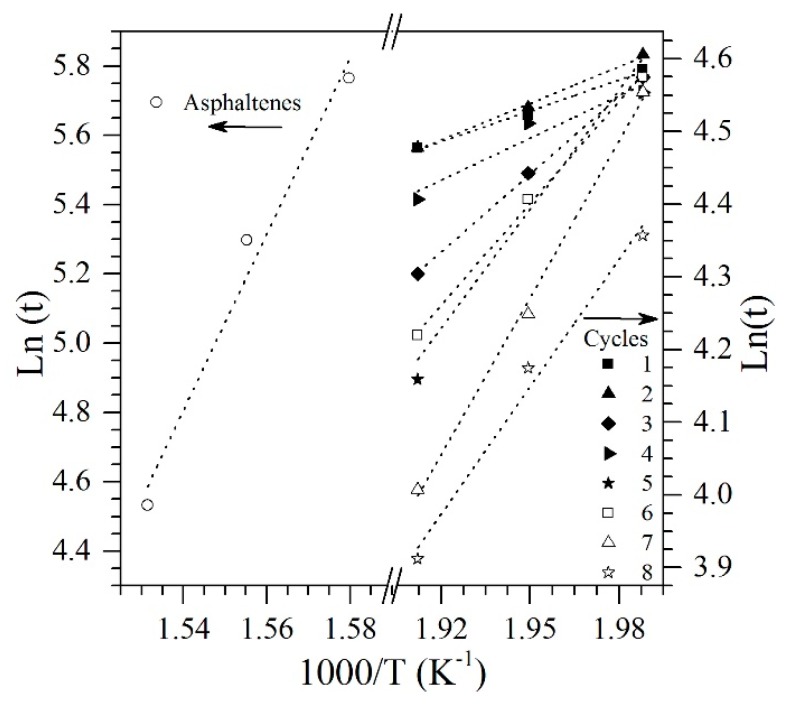
Arrhenius plot for the isothermal model of decomposition-gasification of *n*-C_7_ asphaltenes in the presence and absence of nanoparticles CeNi0.89Pd1.1 through regeneration cycles of adsorption and subsequent catalytic steam gasification.

**Figure 7:**
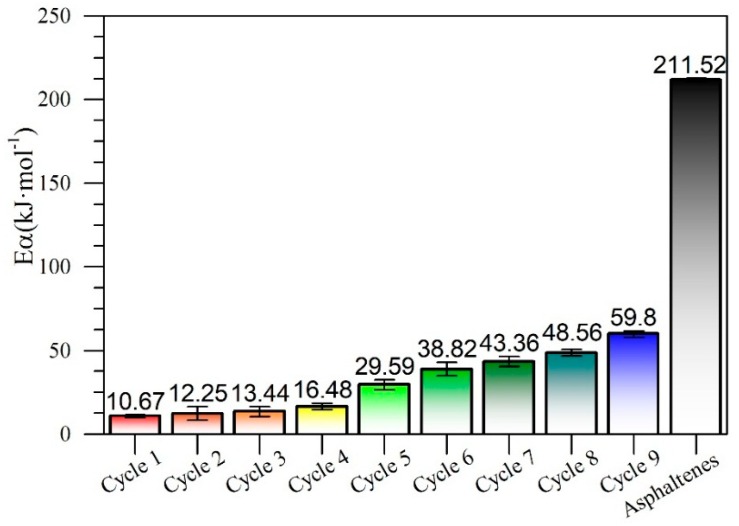
Estimated values of activation energy for isothermal model of catalytic steam decomposition of *n*-C_7_ asphaltenes using CeNi0.89Pd1.1 nanoparticles through catalytic regeneration cycles of adsorption and subsequent catalytic steam gasification.

Effective activation energy was calculated following the isothermal method [69]. For this, plots of $\ln(t_{a,i})$ vs. $1\cdot T_{i}{}^{\;-1}$ were constructed (Figure 6), and the value of the activation energy for each cycle was calculated from the slope of the straight line, taking into account the values obtained at the three temperatures at (230 °C, 240 °C, and 250 °C). Figure 7 shows the activation energy values for the gasification of virgin *n*-C_7_ asphaltenes and their gasification after adsorption on the CeNi0.89Pd1.1 nanoparticle in each regeneration cycle. The estimated value for the activation energy of the *n*-C_7_ asphaltene decomposition reactions on nanoparticles in cycle 1 was 10.7 kJ∙mol^−1^, that is, it decreased by approximately 95% over the value reported in the absence of nanoparticles (211.52 kJ∙mol^−1^). As the nanoparticle was regenerated, the activation energy increased from cycle 1 to cycle 9 by 49.1 kJ∙mol^−1^. It is important to mention at this point, that up to the seventh cycle, the activation energy values are lower than for the value reported for the CeO_2±δ_ support without functionalization, which is 44 kJ∙mol^−1^ [40]. This means that, although the nanoparticle requires more energy for the reactions associated with the gasification process as it is regenerated, it continues to have a better performance thanks to the addition of the transition elements on its surface. Also, it is important to remember that the main advantage of the NiO- and PdO-functionalized ceria nanoparticles lies in the reduction of the temperature for asphaltene decomposition. Also, the ability of the nanoparticles to change its oxidation state from Ce^3+^ to Ce^4+^ in oxidation and reduction conditions allows having a greater number of interactions with the steam molecules and asphaltene heteroatoms, increasing its reaction capacity and catalytic activity. Finally, the change in the activation energy shows that the gasification reaction was affected by the redox cycle Ce^3+^/Ce^4+^ since the concentration of Ce^3+^ ions on the surface of the nanoparticle decreases. Further, considering that these ions are mainly responsible for the catalytic performance of the support, it can be said that the effective activation energy increases as the capacity of the redox cycle is hindered.

### 4.7. XPS Analysis of CeNi0.89Pd1.1 Nanoparticles through Catalytic Regeneration Cycles

**Figure 8:**
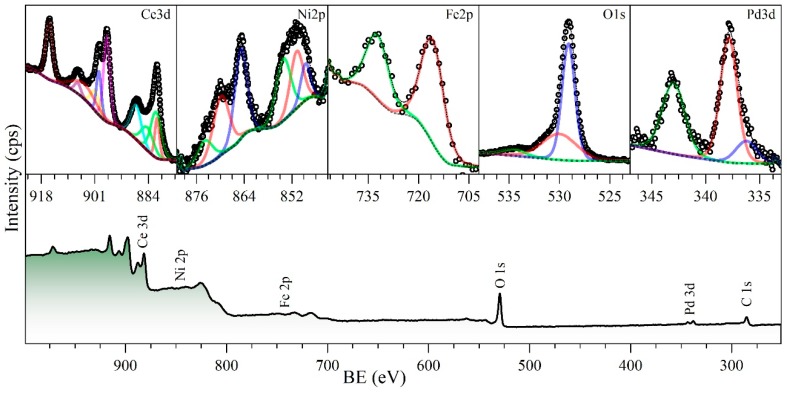
X-ray photoelectron spectroscopy (XPS) spectra of the nanoparticles CeNi0.89Pd1.1 after cycle 4 of regeneration in the *n*-C_7_ asphaltene adsorption/decomposition through catalytic steam gasification for the main elements present at the surface Ce3d, Ni2p, Fe2p, O1s, and Pd3d.

Figure 8 shows the survey spectra for the catalysts after the fourth regeneration cycle. The high-resolution spectra, for the main elements at the surface of the catalyst, are also shown. As expected, the catalyst surface was composed mainly of oxygen from the ceria and the transition elements oxides, palladium, and nickel, as it was nominally prepared (CeNi0.89Pd1.1). Furthermore, iron was found after the first cycle of regeneration. This element proceeds from the asphaltene molecules which could contain some transition elements, such as Fe, Ni, V, depending on their origins [85]. For all samples, carbon from the sample support tape (graphite tape), was analyzed and used as a binding energy reference (284.8 eV). According to the binding energies, after the calcination process of regeneration, all the transition elements are mainly found as oxides. However, as in the O1s high resolution, XPS was decomposed, and around 2% of the superficial oxygen was found as hydroxide. It is well known that Ce compounds were basic, and held the possibility of reacting with water to form cerium hydroxides [86]. Also, Ni and Fe held the same possibility [87,88]. Ni, Fe, and Pd were exhibited on the catalyst surface in the oxides NiO, Fe_2_O_3_, and PdO, respectively. For Pd, a signal at 335.5 eV was present, showing that a fraction of the element was present at the surface in its metallic state (Pd^0^) [89]. This phenomenon can be explained by the thermal decomposition of the PdO to form Pd^0^ and gaseous oxygen [90].

**Figure 9:**
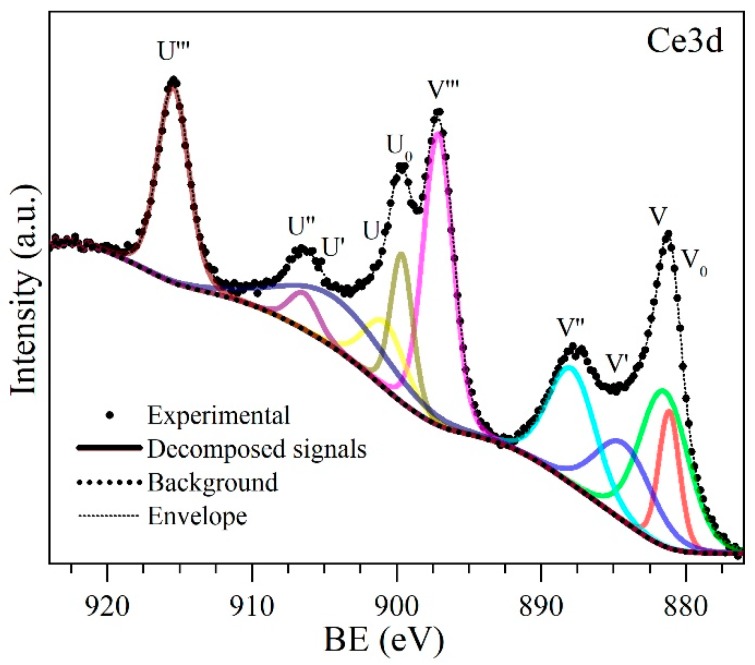
Decomposed high-resolution XPS Ce3d spectrum. U^0^, V^0^, U′, and V′ correspond to Ce^3+^, and U, V, U″, V″, U‴, V‴ to Ce^4+^ [91].

Figure 9 shows the Ce3d decomposed XPS high-resolution spectrum corresponding to cycle 4 and the components of the decomposed signals used to calculate the percentage of $Ce^{3+}$ and $Ce^{4+}$ are indicated [91]. As the cycles occur, the component u, v, u″, v″ and u‴, v‴ (corresponding to $Ce^{4+}$) increase in relation to the $u^{0}$, $v^{0}$ and u′, v′ of the $Ce^{3+}$.

**Figure 10:**
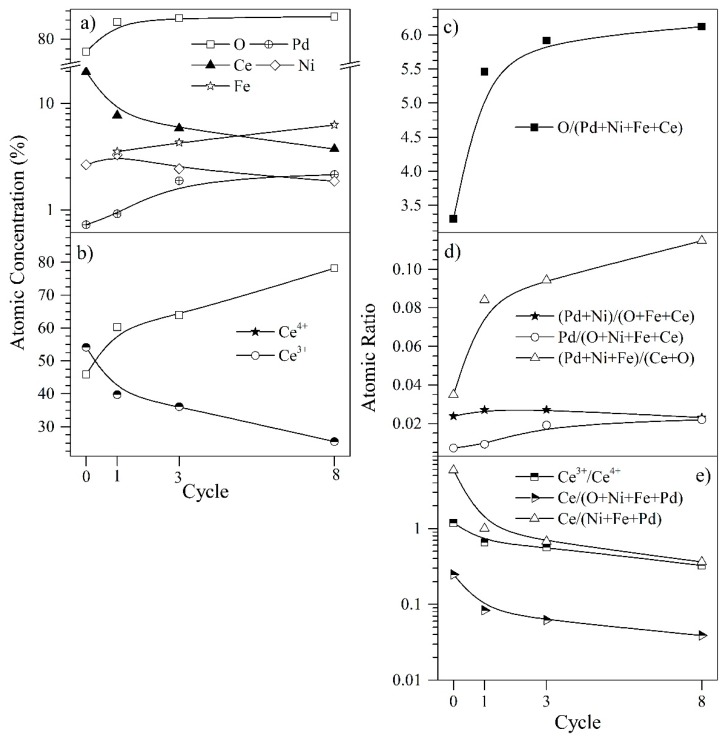
Surface atomic concentration and ratios of the different analyzed elements through XPS of the CeNi0.89Pd1.1 nanoparticles in the *n*-C_7_ asphaltene adsorption/decomposition in catalytic steam gasification for the regeneration cycles 1, 2, 4 and 9. (**a**) Atomic concentration of the all analyzed elements, (**b**) atomic Ce^3+^ and Ce^4+^ concentrations, (**c**,**d**) atomic ratios and (**e**) cerium ratios and concentrations according to oxidation state.

Using the XPS spectra survey at high resolution, the different concentrations and atomic ratios at the surface of the catalyst CeNi0.89Pd1.1 were calculated. These results are shown in Figure 10 as a function of the regeneration cycles (1, 2, 4, y 9). In Figure 10a, the concentrations of the most relevant elements are shown. The surface of the nanoparticles is mainly composed of oxygen that increases its concentration in each regeneration cycle. Conversely, the concentration of ceria on the nanoparticles’ surface decreased with the passage of cycles, indicating that the active sites composed of ceria are covered by heteroatoms such as oxygen and iron.

It is known that ceria signals present quite complex characteristics due to hybridization with bound orbitals and the fractional occupation of valence orbitals 4f [92,93]. It has been shown that the Ce_3d_ XPS spectrum of a $Ce^{4+}$ compound can be deconvoluted into six signals and, if some $Ce^{3+}$ species are also present, four more signals have to be added [93]. From these peaks, it can be observed that the oxidation state $Ce^{4+}$ is predominant while the $Ce^{3+}$ peaks are weak. This is because the electrons are easily transferred from $Ce^{3+}$ to oxygen or other species. The $Ce^{3+}$/$Ce^{4+}$ ratio on the surface of the samples, analyzed over the catalytic regeneration cycles shown in Figure 10c, is strongly affected by the absolute binding energy positions that are characteristic of Ce_3d_. The percentages of $Ce^{3+}$ and $Ce^{4+}$ were calculated using Equations (16) and (17) [94,95,96,97,98,99,100].

(16) $$Ce^{3+}=U^{0}{+U\prime +V}^{0}+V\prime$$

(17) $$Ce^{4+}=U\prime \prime \prime +U\prime \prime +U+V\prime \prime \prime +V\prime \prime +V$$

As mentioned above, the CeO_2±δ_ also has autocatalytic properties that have the ability to reverse the oxidation state from $Ce^{4+}$ to $Ce^{3+}$, allowing it to return to its initial state. However, through the progression of the regeneration cycles of the nanoparticles, the percentage of $Ce^{3+}$ ions present on the surface of the nanoparticle decreases, and since $Ce^{3+}$ ions are responsible for providing CeO_2±δ_ with catalytic activity, the latter decreases too. In addition, the concentration ratio of Ce^4+^/Ce^3+^ ions in the ceria nanoparticles also decreases with the passage of the cycles. Therefore, the number of oxygen vacancies on the surface increased, and this was reflected in the behavior shown in Figure 10c. Consequently, reaction capacity decreases to the extent that the nanoparticle loses part of its catalytic activity due to the interruption in its redox cycle. The concentration ratio of Ce^4+^/Ce^3+^ ions in the ceria nanoparticles is important for the number of oxygen vacancies on the surface, which determine reaction capacity. The mechanism had been described by Equations (18)−(20) [55]:

(18) $$Ce^{3+}\;\to Ce^{4+}+\;e^{-}$$

(19) $$Ce^{3+}+\;OH\to Ce^{4+}+OH^{-}$$

(20) $$Ce^{4+}+\;O_{2}{}^{-}\to Ce^{3+}+\;O_{2}.$$

Figure 10b, however, shows the variation of the transition elements (TE, Ni + Fe + Pd) related to the cerium and oxygen on the surface. It is possible to observe that TE increases during the regeneration cycles, which implies that the cerium atoms are less accessible for the chemical reactions and involved in the redox cycle (Ce^4+^/Ce^3+^) as shown by the decrease in Ce^3+^ concentration in atomic ratio to the Ce^4+^ (see Figure 10c). All these facts imply a decrease of active sites and therefore a reduction of catalytic activity in the nanomaterial. The deposition of the iron over the catalyst’s surface, which increases the TE content, can block the active catalytic sites of the ceria, implying a loss of catalytic activity. Also, the Ni concentration on the surface of the nanoparticle possibly decreases due to a coating of the ceria on the active site of the metal, decreasing its exposed surface area.

#### 4.7.1. Catalytic Effect of Ce^4+^/Ce^3+^ Redox Couple on the Thermodynamic and Adsorption Properties of CeNi0.89Pd1.1 Nanoparticles

**Figure 11:**
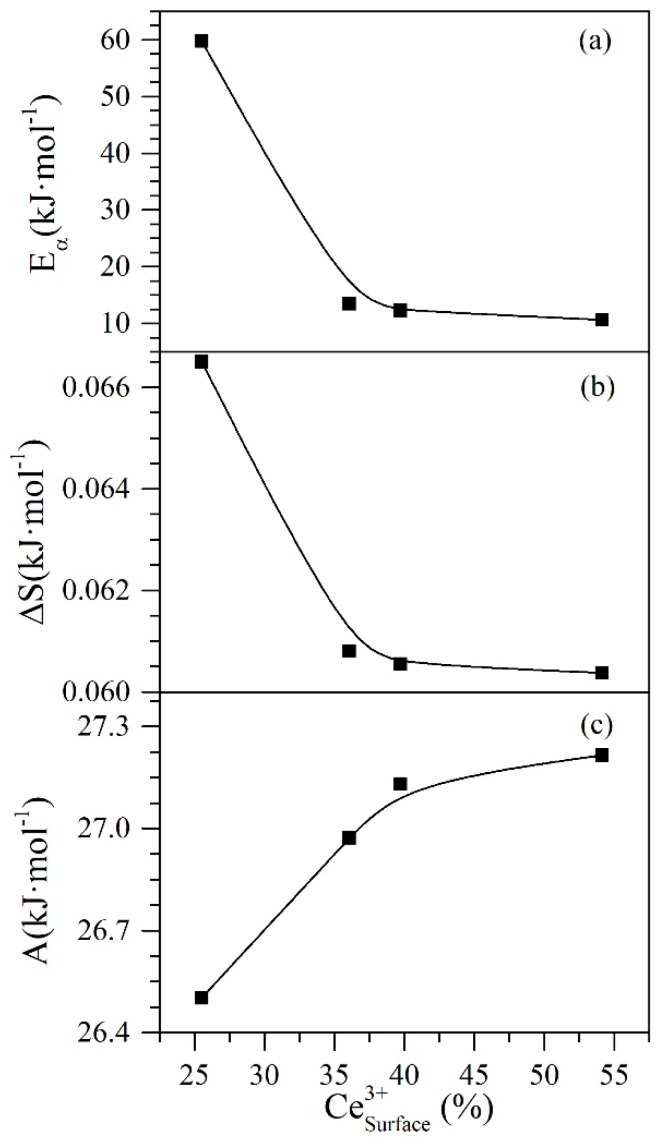
Catalytic effect of $Ce^{3+}$ ions on (**a**) activation energy, (**b**) entropy and (**c**) Polanyi’s adsorption potential for *n*-C_7_ asphaltene adsorption onto CeNi0.89Pd1.1 nanoparticles catalytic steam decomposition through regeneration catalytic cycles 1, 2, 4 and 9.

As the chemical nature of the CeO_2±δ_ nanoparticles can change depending on the amount of $Ce^{3+}$ and $Ce^{4+}$ species present in the system, it is important to analyze how various properties change with the increase or decrease of species content. Figure 11 shows the behavior of entropy, Polanyi’s potential, and activation energy regarding the concentration of $Ce^{3+}$ ions on the nanoparticle surface through different catalytic regeneration cycles.

Panels a and b in Figure 11 show that the behavior of activation energy and entropy increase with the rising concentration of $Ce^{3+}$ ions on the surface of the nanoparticles. This is possibly because these ions are responsible for the catalytic activity of the cerium oxide. The decrease in the presence of ions causes a reduction of this activity, and therefore, the nanoparticles will require more energy for the thermal gasification reactions that decompose the adsorbed heavy fractions. On the contrary and as expected, the Polanyi potential decreases with the reduction in $Ce^{3+}$ ions. If the nanoparticle is losing catalytic activity, it may be due to a reduction in its adsorptive capacity and affinity to adsorb asphaltenes. As explained in Section 4.5, the potential to bring the asphaltene from the bulk phase to the surface of the nanoparticle becomes lower when there is a reduced affinity between the adsorbed-adsorbent pair.

#### 4.7.2. Effective Activation Energy and Kinetics of the Catalytic Steam Gasification of Asphaltenes in the Presence and Absence of Nanoparticles

**Figure 12:**
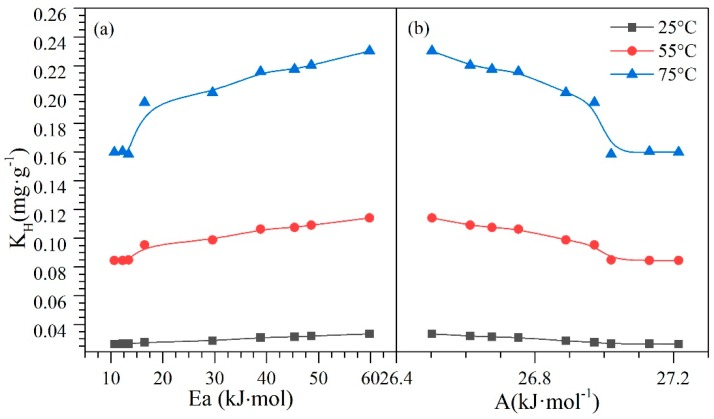
The relationship between Henry’s law constant and (**a**) Polanyi’s adsorption potential and (**b**) effective activation energy for *n*-C_7_ asphaltene adsorption onto CeNi0.89Pd1.1 nanoparticles and subsequent catalytic steam decomposition through regeneration catalytic cycles.

Figure 12 shows the Henry constant as a function of (a) Polanyi’s potential and (b) activation energy. There is a direct relationship between the adsorptive and catalytic properties of the evaluated nanoparticles. From panel a of Figure 12, it can be seen a slight increase in the values of Henry’s law constant as Polanyi’s potential decreases. This behavior is mainly due to the lower affinity between the adsorbate−adsorbent pair, so the potential decreases. Also, this trend is independent of the temperature. Conversely, if this affinity decreases, the energy needed to thermally decompose the asphaltene molecules increases. This behavior can be observed in panel b of Figure 12.

**Figure 13:**
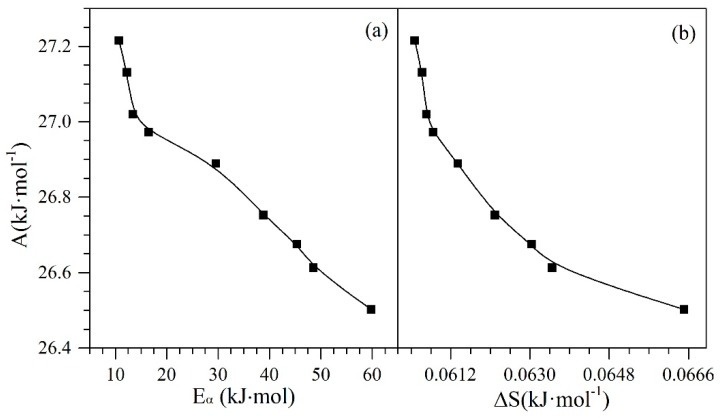
The relationship between Polanyi’s adsorption potential and (**a**) effective activation energy and (**b**) entropy for *n*-C_7_ asphaltene adsorption onto CeNi0.89Pd1.1 nanoparticles and subsequent catalytic steam decomposition through regeneration catalytic cycles.

Finally, Figure 13 shows the behavior of the Polanyi’s potential (*A*) as a function of the activation energy and the change in the entropy of the system. From Figure 13, it is possible to see a decrease in the *A* values from 27.2 kJ∙mol^−1^ to 26.5 kJ∙mol^−1^ as the activation energy and entropy increase. With the decrease in Ce^3+^, the reduction in redox capability, and the increase of the TE on the surface of the nanoparticles with the passage of catalytic regeneration cycles, the catalytic capacity begins to decrease, and consequently, the nanomaterials require more energy to promote water-gas shift and steam reforming reactions. The increase in the entropy change as the adsorption potential decreases is due to the loss of affinity between the adsorbate−adsorbent, generating that the asphaltene presents weaker interactions with nanoparticles, and therefore, interacts more easily with other asphaltene molecules due to their self-assembling capacity. All of these tendencies show how the synergetic interaction between TE (Pd and Ni) and the support (CeO_2±δ_ in this case) are important and crucial for catalytic performance with regard to activation energy and resistance to regeneration through the oxidation cycles. Also, as shown in these results, both the Ce^3+^/Ce^4+^ redox couple and their interaction with the TE are quite important.

## 5. Conclusions

This study provides information on the *n*-C_7_ asphaltene adsorption/decomposition through various cycles of CeO_2±δ_ nanoparticle regeneration. These nanoparticles were functionalized with NiO and PdO (CeNi0.89Pd1.1) to provide them with greater selectivity and catalytic activity. It was demonstrated that the adsorption properties of the nanoparticles do not change significantly as the cycles advance, thus confirming their regenerative capacity. That is to say, the autocatalytic properties of CeO_2±δ_ that allow it to reverse oxidation state also give rise to a new cycle of *n*-C_7_ asphaltene adsorption/decomposition. This is reflected in the adsorption kinetics, where for a fixed concentration of *n*-C_7_ asphaltenes, the amount adsorbed and the time needed to reach equilibrium remain approximately constant. Likewise, the adsorption isotherm nanoparticles were observed to maintain their capacity for *n*-C_7_ asphaltene adsorption, with also a high affinity for the asphaltene to be adsorbed by the nanoparticle. The slight increase in the Polanyi adsorption potential results showed that the work required for an adsorbate molecule to transfer to the surface of the adsorbent does not vary significantly between cycles, which consequently conserves the adsorption properties of nanoparticles.

On the other hand, with the thermodynamic parameters calculated using the SLE model, it was shown that the process is spontaneous and thermodynamically favorable, that is, it does not need additional energy; it also has exothermic nature and randomness in the adsorbent/adsorbate interface. This is reflected in the negative values of the free energy of Gibbs and the enthalpy, and the positive value of entropy. The nanoparticles were able to completely decompose the *n*-C_7_ asphaltenes during all cycles causing a decrease in the activation energy necessary to carry out the asphaltene partial oxidation and gasification reactions. This serves as further proof that the catalytic property is not lost with the passage of the adsorption and decomposition cycles.

Finally, with this study, it was possible to demonstrate that functionalized CeO_2±δ_ nanoparticles may improve the oil recovery process when applied to steam injection processes, allowing greater *n*-C_7_ asphaltene decomposition without losing their catalytic and adsorption properties.

## Figures and Tables

**Table 1:** Estimated values of the parameters of the double exponential model of *n*-C_7_ asphaltene adsorption kinetics on CeNi0.89Pd1.1 nanoparticles through catalyst regeneration cycles of adsorption and subsequent catalytic steam gasification. The parameters are the amount adsorbed ($N_{ads,m,cal}$), the adsorption ($D_{f}$) and mass transfer ($k_{f}$) coefficients for the fast stage, and the adsorption ($D_{s}$) and mass transfer ($k_{s}$) coefficients for the slow stage, respectively. Adsorption kinetics were obtained for an initial *n*-C_7_ asphaltenes concentration of 10 mg·L^−1^.

Cycle	$N_{ads,m,\exp}$ ± 0.01 (mg·g^−1^)	$N_{ads,m,cal}$ ± 0.01 (mg·g^−1^)	$D_{f}$ ± 0.02 (mg·L^−1^)	$k_{f}$ ± 0.01 (min^−1^)	$D_{s}$ ± 0.02 (mg·L^−1^)	$k_{s}$ ± 0.01 (min^−1^)	%RMS
1	0.17	0.17	0.98	0.41	0.45	0.06	0.12
2	0.16	0.17	0.76	0.38	0.34	0.03	0.11
3	0.15	0.15	0.69	0.21	0.65	0.00	0.29
4	0.15	0.15	0.70	0.36	0.20	0.02	0.28
5	0.15	0.15	0.84	0.63	0.47	0.10	0.14
6	0.14	0.14	0.59	0.24	0.97	0.01	0.17
7	0.14	0.13	0.79	0.34	0.73	0.10	0.13
8	0.12	0.12	0.83	0.22	0.12	0.02	0.07
9	0.12	0.12	0.65	0.20	0.53	0.00	0.28

**Table 2:** Estimated values of the solid−liquid equilibrium (SLE model parameters Henry’s law constant ($K_{H}$), the degree of self-association ($K_{.}$) and maximum amount adsorbed ($N_{max}$) for *n*-C_7_ asphaltene adsorption isotherms onto CeNi0.89Pd1.1 nanoparticles, evaluated at 25 °C, 55 °C, and 75 °C through catalyst regeneration of adsorption and subsequent catalytic steam gasification.

Cycle	Temperature	$K_{H}\pm \;0.02$ [mg·g^−1^] × 10^−2^	$K_{.}\pm \;0.08$ [g·g^−1^] × 10^−2^	$N_{max}\pm \;0.01$ [g·g^−1^] × 10^−2^	%RMS
	25	2.64	1.15	27.03	0.004
**1**	55	8.45	3.35	28.86	0.014
	75	15.98	3.45	29.68	0.023
	25	2.65	1.15	27.02	0.004
**2**	55	8.46	3.36	28.57	0.013
	75	16.03	3.46	29.43	0.020
	25	2.65	1.16	25.78	0.004
**3**	55	8.49	3.37	27.19	0.010
	75	15.84	3.48	29.43	0.012
	25	2.76	1.16	25.46	0.002
**4**	55	9.54	3.37	27.13	0.003
	75	19.44	3.48	29.43	0.007
	25	2.86	1.16	25.35	0.001
**5**	55	9.88	3.37	27.02	0.002
	75	20.12	3.49	29.33	0.022
	25	3.09	1.16	25.31	0.000
**6**	55	10.63	3.36	27.01	0.003
	75	21.57	3.48	29.09	0.026
**7**	25	3.15	1.17	24.00	0.000
	55	10.75	3.42	25.91	0.005
	75	21.76	3.49	27.49	0.039
**8**	25	3.20	1.17	22.81	0.001
	55	10.90	3.45	24.50	0.010
	75	22.03	3.49	25.88	0.063
**9**	25	3.35	1.17	22.37	0.006
	55	11.41	3.44	24.03	0.036
	75	23.03	3.54	25.36	0.119

**Table 3:** Thermodynamic parameters for the adsorption of *n*-C_7_ asphaltene onto CeNi0.89Pd1.1 nanoparticles through different catalyst regeneration cycles of adsorption and subsequent catalytic steam gasification. The change in entropy is expressed by $\Delta S_{ads}^{o}$, change in enthalpy is $\Delta H_{ads}^{o}$ and change in Gibbs free energy is $\Delta G_{ads}^{o}$.

Cycle	Temperature (°C)	$\Delta S_{ads}^{o}$ ± 0.02 × 10^−2^ [J·(mol·K)^−1^]	$-\Delta H_{ads}^{o}$ ± 0.01 [kJ·mol^−1^]	$-\Delta G_{ads}^{o}$ ± 0.01 [J·mol^−1^]
	25			6.04
**1**	55	6.03	29.08	9.58
	75			10.25
**2**	25	6.05	29.13	6.04
	55			9.58
	75			9.90
**3**	25	6.07	29.18	6.03
	55			9.57
	75			9.68
**4**	25	6.08	29.23	6.02
	55			9.56
	75			9.67
	25	6.14	29.32	6.02
**5**	55			9.56
	75			9.66
	25	6.22	29.36	6.02
**6**	55			9.55
	75			9.63
	25	6.30	29.43	6.02
**7**	55			9.54
	75			9.60
	25	6.35	29.66	6.02
**8**	55			9.53
	75			9.60
	25	6.65	29.87	6.01
**9**	55			9.28
	75			9.58

## References

[1] Shah A., Fishwick R., Wood J., Leeke G., Rigby S., Greaves M. (2010). A review of novel techniques for heavy oil and bitumen extraction and upgrading. Energy Environ. Sci..

[2] International Energy Agency (2017). World Energy Outlook 2017.

[3] Santos R., Loh W., Bannwart A., Trevisan O. (2014). An overview of heavy oil properties and its recovery and transportation methods. Braz. J. Chem. Eng..

[4] Guo K., Li H., Yu Z. (2016). In-situ heavy and extra-heavy oil recovery: A review. Fuel.

[5] Curtis C., Kopper R., Decoster E., Guzmán-Garcia A., Huggins C., Knauer L., Minner M., Kupsch N., Linares L.M., Rough H. (2002). Heavy-oil reservoirs. Oilfield Rev..

[6] Rahimi P.M., Gentzis T. (2006). The chemistry of bitumen and heavy oil processing. Practical Advances in Petroleum Processing.

[7] Hamedi Shokrlu Y., Babadagli T. (2014). Kinetics of the in-situ upgrading of heavy oil by nickel nanoparticle catalysts and its effect on cyclic-steam-stimulation recovery factor. SPE Reserv. Eval. Eng..

[8] Nassar N.N., Hassan A., Pereira-Almao P. (2011). Metal oxide nanoparticles for asphaltene adsorption and oxidation. Energy Fuels.

[9] Buckley J.S., Liu Y., Xie X., Morrow N.R. (1997). Asphaltenes and crude oil wetting-the effect of oil composition. SPE J..

[10] Terry R.E. (2001). Enhanced oil recovery. Encycl. Phys. Sci. Technol..

[11] Alvarado V., Manrique E. (2010). Enhanced oil recovery: An update review. Energies.

[12] Thomas S. (2008). Enhanced oil recovery—An overview. Oil Gas Sci. Technol. Revue de l’IFP.

[13] Cardona L., Arias-Madrid D., Cortés F., Lopera S., Franco C. (2018). Heavy oil upgrading and enhanced recovery in a steam injection process assisted by NiO-and PdO-Functionalized SiO_2_ nanoparticulated catalysts. Catalysts.

[14] Wang Y., Ren S., Zhang L., Peng X., Pei S., Cui G., Liu Y. (2018). Numerical study of air assisted cyclic steam stimulation process for heavy oil reservoirs: Recovery performance and energy efficiency analysis. Fuel.

[15] Chaar M., Venetos M., Dargin J., Palmer D. (2015). Economics of Steam Generation for Thermal Enhanced Oil Recovery. Oil Gas Facil..

[16] Ali S., Thomas S. (1996). The promise and problems of enhanced oil recovery methods. J. Can. Pet. Technol..

[17] Karacan C.Ö., Okandan E. (1997). Change of physical and thermal decomposition properties of in situ heavy oil with steam temperature. Pet. Sci. Technol..

[18] Kowalewski I., Fiedler C., Parra T., Adam P., Albrecht P. (2008). Preliminary results on the formation of organosulfur compounds in sulfate-rich petroleum reservoirs submitted to steam injection. Org. Geochem..

[19] Kar T., Ovalles C., Rogel E., Vien J., Hascakir B. (2016). The residual oil saturation determination for Steam Assisted Gravity Drainage (SAGD) and Solvent-SAGD. Fuel.

[20] Nassar N.N., Franco C.A., Montoya T., Cortés F.B., Hassan A. (2015). Effect of oxide support on Ni–Pd bimetallic nanocatalysts for steam gasification of n-C7 asphaltenes. Fuel.

[21] Nassar N.N., Hassan A., Pereira-Almao P. (2011). Application of nanotechnology for heavy oil upgrading: Catalytic steam gasification/cracking of asphaltenes. Energy Fuels.

[22] Sun X., Zhang Y., Chen G., Gai Z. (2017). Application of nanoparticles in enhanced oil recovery: A critical review of recent progress. Energies.

[23] Agista M.N., Guo K., Yu Z. (2018). A State-of-the-Art Review of Nanoparticles Application in Petroleum with a Focus on Enhanced Oil Recovery. Appl. Sci..

[24] Hashemi R., Nassar N.N., Almao P.P. (2014). Nanoparticle technology for heavy oil in-situ upgrading and recovery enhancement: Opportunities and challenges. Appl. Energy.

[25] Cheraghian G., Hendraningrat L. (2016). A review on applications of nanotechnology in the enhanced oil recovery part B: Effects of nanoparticles on flooding. Int. Nano Lett..

[26] Franco C.A., Montoya T., Nassar N.N., Pereira-Almao P., Cortés F.B. (2013). Adsorption and subsequent oxidation of colombian asphaltenes onto nickel and/or palladium oxide supported on fumed silica nanoparticles. Energy Fuels.

[27] López D., Giraldo L.J., Salazar J.P., Zapata D.M., Ortega D.C., Franco C.A., Cortés F.B. (2017). Metal Oxide Nanoparticles Supported on Macro-Mesoporous Aluminosilicates for Catalytic Steam Gasification of Heavy Oil Fractions for On-Site Upgrading. Catalysts.

[28] Hosseinpour N., Mortazavi Y., Bahramian A., Khodatars L., Khodadadi A.A. (2014). Enhanced pyrolysis and oxidation of asphaltenes adsorbed onto transition metal oxides nanoparticles towards advanced in-situ combustion EOR processes by nanotechnology. Appl. Catal. A Gen..

[29] Nassar N.N., Hassan A., Pereira-Almao P. (2011). Comparative oxidation of adsorbed asphaltenes onto transition metal oxide nanoparticles. Colloids Surf. A Physicochem. Eng. Asp..

[30] Nassar N.N., Hassan A., Carbognani L., Lopez-Linares F., Pereira-Almao P. (2012). Iron oxide nanoparticles for rapid adsorption and enhanced catalytic oxidation of thermally cracked asphaltenes. Fuel.

[31] Nassar N.N., Hassan A., Pereira-Almao P. (2011). Effect of the particle size on asphaltene adsorption and catalytic oxidation onto alumina particles. Energy Fuels.

[32] Druetta P., Raffa P., Picchioni F. (2018). Plenty of Room at the Bottom: Nanotechnology as Solution to an Old Issue in Enhanced Oil Recovery. Appl. Sci..

[33] Hassan A., Lopez-Linares F., Nassar N.N., Carbognani-Arambarri L., Pereira-Almao P. (2013). Development of a support for a NiO catalyst for selective adsorption and post-adsorption catalytic steam gasification of thermally converted asphaltenes. Catal. Today.

[34] Sehested J., Gelten J.A., Remediakis I.N., Bengaard H., Nørskov J.K. (2004). Sintering of nickel steam-reforming catalysts: Effects of temperature and steam and hydrogen pressures. J. Catal..

[35] Nezhad S.S.K., Cheraghian G. (2016). Mechanisms behind injecting the combination of nano-clay particles and polymer solution for enhanced oil recovery. Appl. Nanosci..

[36] Cheraghian G., Nezhad S.S.K., Kamari M., Hemmati M., Masihi M., Bazgir S. (2014). Adsorption polymer on reservoir rock and role of the nanoparticles, clay and SiO2. Int. Nano Lett..

[37] Cheraghian G. (2017). Synthesis and properties of polyacrylamide by nanoparticles, effect nanoclay on stability polyacrylamide solution. Micro Nano Lett..

[38] Cheraghian G. (2017). Evaluation of clay and fumed silica nanoparticles on adsorption of surfactant polymer during enhanced oil recovery. J. Jpn. Pet. Inst..

[39] Cardona Rojas L. (2018). Efecto de nanopartículas en procesos con inyección de vapor a diferentes calidades. Master’s Thesis.

[40] Mdina O.E., Gallego J., Arias-Madrid D., Cortés F.B., Franco C.A. (2019). Optimization of the Load of Transition Metal Oxides (Fe_2_O_3_, Co_3_O_4_, NiO and/or PdO) onto CeO_2_ Nanoparticles in Catalytic Steam Decomposition of n-C7 Asphaltenes at Low Temperatures. Nanomaterials.

[41] Atta A., Al-Lohedan H., Al-Hussain S. (2015). Functionalization of magnetite nanoparticles as oil spill collector. Int. J. Mol. Sci..

[42] Guo K., Zhang Y., Shi Q., Yu Z. (2017). The effect of carbon-supported nickel nanoparticles in the reduction of carboxylic acids for in situ upgrading of heavy crude oil. Energy Fuels.

[43] Hosseinpour M., Fatemi S., Ahmadi S.J. (2015). Catalytic cracking of petroleum vacuum residue in supercritical water media: Impact of α-Fe_2_O_3_ in the form of free nanoparticles and silica-supported granules. Fuel.

[44] Hosseinpour N., Khodadadi A.A., Bahramian A., Mortazavi Y. (2013). Asphaltene adsorption onto acidic/basic metal oxide nanoparticles toward in situ upgrading of reservoir oils by nanotechnology. Langmuir.

[45] Kazemzadeh Y., Eshraghi S.E., Kazemi K., Sourani S., Mehrabi M., Ahmadi Y. (2015). Behavior of asphaltene adsorption onto the metal oxide nanoparticle surface and its effect on heavy oil recovery. Ind. Eng. Chem. Res..

[46] Li L., Zhan Y., Zheng Q., Zheng Y., Lin X., Li D., Zhu J. (2007). Water–Gas Shift Reaction Over Aluminum Promoted Cu/CeO2 Nanocatalysts Characterized by XRD, BET, TPR and Cyclic Voltammetry (CV). Catal. Lett..

[47] Nematollahi B., Rezaei M., Lay E.N. (2015). Preparation of highly active and stable NiO–CeO2 nanocatalysts for CO selective methanation. Int. J. Hydrog. Energy.

[48] Khajenoori M., Rezaei M., Nematollahi B. (2013). Preparation of noble metal nanocatalysts and their applications in catalytic partial oxidation of methane. J. Ind. Eng. Chem..

[49] Estifaee P., Haghighi M., Mohammadi N., Rahmani F. (2014). CO oxidation over sonochemically synthesized Pd–Cu/Al_2_O_3_ nanocatalyst used in hydrogen purification: Effect of Pd loading and ultrasound irradiation time. Ultrason. Sonochem..

[50] De Lasa H., Salaices E., Mazumder J., Lucky R. (2011). Catalytic steam gasification of biomass: Catalysts, thermodynamics and kinetics. Chem. Rev..

[51] Koh A.C., Leong W.K., Chen L., Ang T.P., Lin J., Johnson B.F., Khimyak T. (2008). Highly efficient ruthenium and ruthenium–platinum cluster-derived nanocatalysts for hydrogen production via ethanol steam reforming. Catal. Commun..

[52] Vidal H., Kašpar J., Pijolat M., Colon G., Bernal S., Cordón A., Perrichon V., Fally F. (2000). Redox behavior of CeO2–ZrO2 mixed oxides: I. Influence of redox treatments on high surface area catalysts. Appl. Catal. B Environ..

[53] Wang R., Xu H., Liu X., Ge Q., Li W. (2006). Role of redox couples of Rh0/Rhδ+ and Ce4+/Ce3+ in CH4/CO2 reforming over Rh–CeO_2_/Al_2_O_3_ catalyst. Appl. Catal. A Gen..

[54] Celardo I., Traversa E., Ghibelli L. (2011). Cerium oxide nanoparticles: A promise for applications in therapy. J. Exp. Ther. Oncol..

[55] Das M., Patil S., Bhargava N., Kang J.-F., Riedel L.M., Seal S., Hickman J.J. (2007). Auto-catalytic ceria nanoparticles offer neuroprotection to adult rat spinal cord neurons. Biomaterials.

[56] Hayek K., Kramer R., Paál Z. (1997). Metal-support boundary sites in catalysis. Appl. Catal. A Gen..

[57] Alamolhoda S., Vitale G., Hassan A., Nassar N.N., Almao P.P. (2019). Synergetic effects of cerium and nickel in Ce-Ni-MFI catalysts on low-temperature water-gas shift reaction. Fuel.

[58] Munnik P., de Jongh P.E., de Jong K.P. (2015). Recent developments in the synthesis of supported catalysts. Chem. Rev..

[59] Lensveld D.J., Mesu J.G., van Dillen A.J., de Jong K.P. (2000). The application of well-dispersed nickel nanoparticles inside the mesopores of MCM-41 by use of a nickel citrate chelate as precursor. Stud. Surf. Sci. Catal..

[60] Mansfield E., Tyner K.M., Poling C.M., Blacklock J.L. (2014). Determination of nanoparticle surface coatings and nanoparticle purity using microscale thermogravimetric analysis. Anal. Chem..

[61] Qiu H., Lv L., Pan B.-C., Zhang Q.-J., Zhang W.-M., Zhang Q.-X. (2009). Critical review in adsorption kinetic models. J. Zhejiang Univ. Sci. A.

[62] Chiron N., Guilet R., Deydier E. (2003). Adsorption of Cu(II) and Pb(II) onto a grafted silica: Isotherms and kinetic models. Water Res..

[63] Wilczak A., Keinath T.M. (1993). Kinetics of sorption and desorption of copper (II) and lead (II) on activated carbon. Water Environ. Res..

[64] Talu O., Meunier F. (1996). Adsorption of associating molecules in micropores and application to water on carbon. AIChE J..

[65] Montoya T., Coral D., Franco C.A., Nassar N.N., Cortés F.B. (2014). A novel solid–liquid equilibrium model for describing the adsorption of associating asphaltene molecules onto solid surfaces based on the “chemical theory”. Energy Fuels.

[66] Nassar N.N. (2010). Asphaltene adsorption onto alumina nanoparticles: Kinetics and thermodynamic studies. Energy Fuels.

[67] Cortés F.B., Montoya T., Acevedo S., Nassar N.N., Franco C.A. (2016). Adsorption-desorption of n-c7 asphaltenes over micro-and nanoparticles of silica and its impact on wettability alteration. CT F-Cienc. Tecnol. Futuro.

[68] Betancur S., Carrasco-Marín F., Franco C.A., Cortés F.B. (2018). Development of Composite Materials Based on the Interaction between Nanoparticles and Surfactants for Application on Chemical Enhanced Oil Recovery. Ind. Eng. Chem. Res..

[69] Nassar N.N., Hassan A., Luna G., Pereira-Almao P. (2013). Kinetics of the catalytic thermo-oxidation of asphaltenes at isothermal conditions on different metal oxide nanoparticle surfaces. Catal. Today.

[70] Vyazovkin S., Burnham A.K., Criado J.M., Pérez-Maqueda L.A., Popescu C., Sbirrazzuoli N. (2011). ICTAC Kinetics Committee recommendations for performing kinetic computations on thermal analysis data. Thermochim. Acta.

[71] Scheffe H. (1963). The simplex-centroid design for experiments with mixtures. J. R. Stat. Soc. Ser. B.

[72] Murugan P., Mahinpey N., Mani T. (2009). Thermal cracking and combustion kinetics of asphaltenes derived from Fosterton oil. Fuel Process. Technol..

[73] Mullins O.C., Sabbah H., Eyssautier J., Pomerantz A.E., Barré L., Andrews A.B., Ruiz-Morales Y., Mostowfi F., McFarlane R., Goual L. (2012). Advances in asphaltene science and the Yen–Mullins model. Energy Fuels.

[74] Nassar N.N., Betancur S., Acevedo S.c., Franco C.A., Cortés F.B. (2015). Development of a population balance model to describe the influence of shear and nanoparticles on the aggregation and fragmentation of asphaltene aggregates. Ind. Eng. Chem. Res..

[75] Franco C., Patiño E., Benjumea P., Ruiz M.A., Cortés F.B. (2013). Kinetic and thermodynamic equilibrium of asphaltenes sorption onto nanoparticles of nickel oxide supported on nanoparticulated alumina. Fuel.

[76] Wu S., Tang D., Li S., Chen H., Wu H. (2016). Coalbed methane adsorption behavior and its energy variation features under supercritical pressure and temperature conditions. J. Pet. Sci. Eng..

[77] Franco C.A., Nassar N.N., Montoya T., Ruíz M.A., Cortés F.B. (2015). Influence of asphaltene aggregation on the adsorption and catalytic behavior of nanoparticles. Energy Fuels.

[78] Sharma A., Saito I., Nakagawa H., Miura K. (2007). Effect of carbonization temperature on the nickel crystallite size of a Ni/C catalyst for catalytic hydrothermal gasification of organic compounds. Fuel.

[79] Cao A., Lu R., Veser G. (2010). Stabilizing metal nanoparticles for heterogeneous catalysis. Phys. Chem. Chem. Phys..

[80] Arboleda J., Castillo Á., Muñoz S. (2018). Estudio de la acuatermólisis catalítica en procesos de upgrading de crudos pesados como método complementario en el recobro térmico de hidrocarburos. Rev. Fuentes.

[81] Ospina Gómez N.A. (2015). Evaluación de la aplicación de nanofluidos para mejoramiento in-situ del crudo pesado. Master’s Thesis.

[82] Ternan M. (1983). Catalytic hydrogenation and asphaltene conversion of Athabasca bitumen. Can. J. Chem. Eng..

[83] Jacobs G., Ricote S., Graham U.M., Patterson P.M., Davis B.H. (2005). Low temperature water gas shift: Type and loading of metal impacts forward decomposition of pseudo-stabilized formate over metal/ceria catalysts. Catal. Today.

[84] Vignatti C.I., Avila M.S., Apesteguia C.R., Garetto T.F. (2011). Study of the water-gas shift reaction over Pt supported on CeO_2_–ZrO_2_ mixed oxides. Catal. Today.

[85] Speight J. (2004). Petroleum Asphaltenes-Part 1: Asphaltenes, resins and the structure of petroleum. Oil Gas Sci. Technol..

[86] Fu Q., Deng W., Saltsburg H., Flytzani-Stephanopoulos M. (2005). Activity and stability of low-content gold–cerium oxide catalysts for the water–gas shift reaction. Appl. Catal. B Environ..

[87] Hilaire S., Wang X., Luo T., Gorte R., Wagner J. (2001). A comparative study of water-gas-shift reaction over ceria supported metallic catalysts. Appl. Catal. A Gen..

[88] Mierczynski P., Maniukiewicz W., Maniecki T. (2013). Comparative studies of Pd, Ru, Ni, Cu/ZnAl2O4 catalysts for the water gas shift reaction. Open Chem..

[89] Peuckert M. (1985). XPS study on surface and bulk palladium oxide, its thermal stability, and a comparison with other noble metal oxides. J. Phys. Chem..

[90] Basile F., Fornasari G., Gazzano M., Vaccari A. (2001). Thermal evolution and catalytic activity of Pd/Mg/Al mixed oxides obtained from a hydrotalcite-type precursor. Appl. Clay Sci..

[91] Pinc W., Yu P., O’Keefe M., Fahrenholtz W. (2009). Effect of gelatin additions on the corrosion resistance of cerium based conversion coatings spray deposited on Al 2024-T3. Surf. Coat. Technol..

[92] Shyu J., Weber W., Gandhi H. (1988). Surface characterization of alumina-supported ceria. J. Phys. Chem..

[93] Abi-aad E., Bechara R., Grimblot J., Aboukais A. (1993). Preparation and characterization of ceria under an oxidizing atmosphere. Thermal analysis, XPS, and EPR study. Chem. Mater..

[94] Matta J., Courcot D., Abi-Aad E., Aboukais A. (2001). Thermal analysis, EPR and XPS study of vanadyl (IV) oxalate behavior on the ceria surface. J. Therm. Anal. Calorim..

[95] Qiu L., Liu F., Zhao L., Ma Y., Yao J. (2006). Comparative XPS study of surface reduction for nanocrystalline and microcrystalline ceria powder. Appl. Surf. Sci..

[96] Sim K.S., Hilaire L., Le Normand F., Touroude R., Paul-Boncour V., Percheron-Guegan A. (1991). Catalysis by palladium–rare-earth-metal (REPd 3) intermetallic compounds: Hydrogenation of but-1-ene, buta-1, 3-diene and but-1-yne. J. Chem. Soc. Faraday Trans..

[97] Kotani A., Jo T., Parlebas J. (1988). Many-body effects in core-level spectroscopy of rare-earth compounds. Adv. Phys..

[98] Laachir A., Perrichon V., Badri A., Lamotte J., Catherine E., Lavalley J.C., El Fallah J., Hilaire L., Le Normand F., Quéméré E. (1991). Reduction of CeO_2_ by hydrogen. Magnetic susceptibility and Fourier-transform infrared, ultraviolet and X-ray photoelectron spectroscopy measurements. J. Chem. Soc. Faraday Trans..

[99] Nagai Y., Hirabayashi T., Dohmae K., Takagi N., Minami T., Shinjoh H., Matsumoto S.i. (2006). Sintering inhibition mechanism of platinum supported on ceria-based oxide and Pt-oxide–support interaction. J. Catal..

[100] Xu J., Harmer J., Li G., Chapman T., Collier P., Longworth S., Tsang S.C. (2010). Size dependent oxygen buffering capacity of ceria nanocrystals. Chem. Commun..

